# Distribution of Flavan-3-ol Species in Ripe Strawberry Fruit Revealed by Matrix-Assisted Laser Desorption/Ionization-Mass Spectrometry Imaging

**DOI:** 10.3390/molecules25010103

**Published:** 2019-12-26

**Authors:** Hirofumi Enomoto, Senji Takahashi, Shiro Takeda, Hajime Hatta

**Affiliations:** 1 Department of Biosciences, Faculty of Science and Engineering, Teikyo University, Utsunomiya 320-8551, Japan; takahasi@nasu.bio.teikyo-u.ac.jp; 2 Division of Integrated Science and Engineering, Graduate School of Science and Engineering, Teikyo University, Utsunomiya 320-8551, Japan; 3 Advanced Instrumental Analysis Center, Teikyo University, Utsunomiya 320-8551, Japan; 4 Department of Animal Science and Biotechnology, School of Veterinary Medicine, Azabu University, Sagamihara 252-5201, Japan; s-takeda@azabu-u.ac.jp; 5 Department of Food and Nutrition, Faculty of Home Economics, Kyoto Women’s University, Kyoto 605-8501, Japan; hatta@kyoto-wu.ac.jp

**Keywords:** strawberry, flavan-3-ols, proanthocyanidins, procyanidins, propelargonidins, mass spectrometry imaging (MSI), matrix-assisted laser desorption/ionization (MALDI)

## Abstract

Flavan-3-ols, which comprise proanthocyanidins and their monomers, are major flavonoids in strawberries, and they have a wide range of biological activities and health benefits. However, their spatial distribution in strawberry fruit remains poorly understood. Therefore, we performed matrix-assisted laser desorption/ionization-mass spectrometry imaging (MALDI-MSI), to visualize flavan-3-ols in ripe strawberry fruit. Peaks matching the *m*/*z* values of flavan-3-ols [M − H]^−^ ions were detected in the negative ion mode using 1,5-diaminonaphthalene as matrix. Catechin and/or epicatechin, three B-type procyanidins, and two B-type propelargonidins were identified by MALDI-tandem MS. These flavan-3-ols were mainly distributed in the calyx, in and around the vascular bundles, and in the skin. In-source fragmentation of proanthocyanidins was determined using their standards, suggesting their distribution was mixed ion images of themselves, and fragment ions generated from those had a higher degree of polymerization. B-type procyanidins were predominantly distributed in the vascular bundles than in the skin, whereas B-type propelargonidins were almost equally distributed between the vascular bundles and skin, suggesting that their distribution patterns are different from the type of their flavan-3-ol monomers. Flavan-3-ols, especially B-type procyanidins, may help prevent pathogen infection not only in the skin but also in and around the vascular bundles.

## 1. Introduction

Proanthocyanidins, also known as condensed tannins, are oligomers and polymers of flavan-3-ol units that have been used as therapeutic compounds since ancient times [1,2]. Flavan-3-ols are one of the most ubiquitous groups of plant phenolics; they are synthesized via the phenylpropanoid and flavonoid pathways [1,2,3] and are distributed widely in plants. Flavan-3-ols also have diverse biological activities, including protection against herbivorous animals and pathogen attacks (both bacterial and fungal), and are effective in restricting the growth of neighboring plants [1]. Flavan-3-ols are also widely distributed in human food of plant origin, particularly fruits, legume seeds, and cereal grains; where, they contribute to the bitter flavor and astringency [1]. In recent years, considerable attention has been paid to proanthocyanidins and their monomers owing to their potential benefits to human health, such as their immunomodulatory and anticancer activities, antioxidant and radical-scavenging functions, anti-inflammatory activities, cardio-protective properties, vasodilating and antithrombotic effects, and protective functions against ultraviolet radiation [3,4].

**Figure 1:**
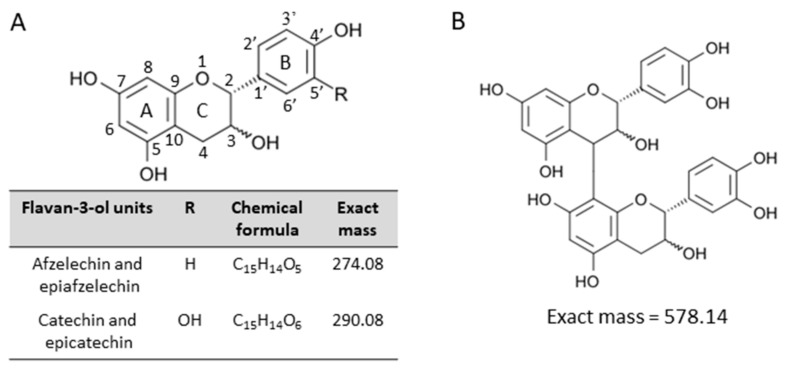
Structures of the flavan-3-ol species analyzed in this study. Structures of (**A**) flavan-3-ol units of proanthocyanidin species, (**B**) B-type procyanidin, (epi)catechin dimer. (Epi)catechin represents either catechin or epicatechin.

Proanthocyanidins consisting exclusively of catechin and/or epicatechin are known as procyanidins, whereas those containing afzelechin and/or epiafzelechin are called propelargonidins [1,5,6,7,8] (Figure 1A). Proanthocyanidins are also categorized as A-type and B-type proanthocyanidins, depending on the linkages between the monomeric flavan-3-ol subunits [1,5,6,7,8,9]. B-type proanthocyanidins are characterized by a single carbon–carbon interflavan bond between the C-ring of one subunit and the A-ring of the next (C4 → C8 or C4 → C6) (Figure 1B), whereas A-type proanthocyanidins have both a carbon–carbon interflavan bond and an additional carbon–oxygen linkage (C2 → O7) [1,5,6,7,8,9].

Strawberries (*Fragaria × ananassa*) are the most widespread and consumed fruits in the world owing to their attractive appearance, taste, and health-related properties [10,11]. Strawberries are rich in nutritious compounds, including, vitamins, and organic acids, as well as non-nutritious phytochemicals, especially polyphenols [10,11]. Data from several studies using strawberry fruit powder or extracts suggested that these nutritious compounds promote beneficial health effects, such as preventative functions against cancer [10,11,12,13,14]. Flavan-3-ols, namely proanthocyanidins and their monomers, are the second-most abundant polyphenols after anthocyanins in strawberry fruit [11]. Thus, in addition to other classes of metabolites, flavan-3-ols are also believed to be associated with the health properties of strawberry fruit. However, the spatial distributions of flavan-3-ols in the bodies of strawberry fruit have not been fully determined.

Mass spectrometry imaging (MSI) is an emerging technique that can be used to simultaneously investigate both the content and spatial distribution of biomolecules, such as metabolites and proteins, in tissues, without requiring antibodies, staining, or complicated preliminary procedures [15,16,17,18,19]. Recently, MSI was adapted for identifying and quantitating various metabolites in food products, which enabled visualization at microscopic resolution using soft ionization techniques, mainly matrix-assisted laser desorption/ionization (MALDI) [20,21,22,23,24,25,26,27] or desorption electrospray ionization [28,29,30]. For example, in our previous study using MALDI-MSI, anthocyanin species such as pelargonidin- and cyanidin-glycosides were successfully visualized and differences in their distribution patterns in strawberry fruit were revealed [24].

In this study, using ripe ‘Tochiotome’ strawberries (a popular strawberry in Japan), we identified B-type propelargonidin and procyanidin species using MALDI-MS/MS analysis. Subsequently, we visualized the spatial distributions of the identified proanthocyanidins and showed their unique distribution by MALDI-MSI analysis. We expect that these results will contribute to further elucidating the biological roles of flavan-3-ol species.

## 2. Results

### 2.1. Matrix Selection for MALDI-MSI Analysis of Flavan-3-ols

To detect proanthocyanidins and their monomers, we initially performed matrix screening using the following matrices: α-cyano-4-hydroxycinnamic acid (CHCA), 9-aminoacridine (9AA), and 1,5-diaminonaphthalene (DAN), which are commonly used in MALDI-MSI. The negative-ion mode of MALDI-MSI was selected over the positive-ion mode because the proanthocyanidin signals were concentrated into one abundant deprotonated molecule, [M − H]^−^ ion, instead of being distributed among mixtures of [M + H]^+^, [M + Na]^+^, and [M + K]^+^ adducts [7,8]. By performing MALDI-MS in the negative-ion mode, we detected afzelechin, catechin, epicatechin, procyanidin B1 (a B-type epicatechin-catechin), B2 (a B-type epicatechin dimer), procyanidin C1 (a B-type epicatechin trimer), and cinnamtannin A2 (a B-type epicatechin tetramer) standards as [M − H]^−^ ions, using all the matrices mentioned above. The maximum signal intensities were achieved using DAN (Supplementary Figure S1). Therefore, in the subsequent experiments, we analyzed flavan-3-ols in strawberry fruit in negative-ion mode using DAN as the matrix.

### 2.2. Mass Spectra Obtained from Strawberry Fruit Sections

**Figure 2:**
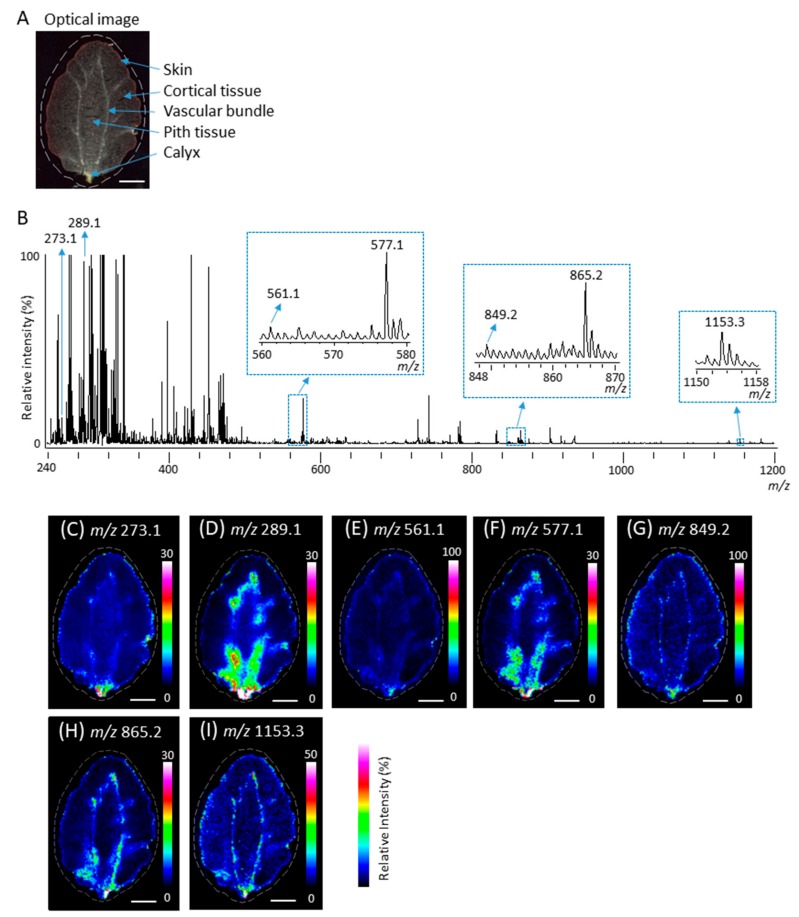
Matrix-assisted laser desorption/ionization (MALDI)-mass spectrometry imaging (MSI) of strawberry fruit sections. (**A**) Optical image of a strawberry fruit section before MALDI-MSI analysis. The dotted white line shows the analyzed region. (**B**) Mass spectrum obtained from the strawberry fruit section. The *m*/*z* values indicated that the peaks were possibly attributable to flavan-3-ols. The relative intensity of the strongest *m*/*z* 289.1 in the peaks with potential compounds of (epi)catechin was defined as 95%. (Epi)catechin represents either catechin or epicatechin. Representative ion images at *m*/*z* (**C**) 273.1, (**D**) 289.1, (**E**) 561.1, (**F**) 577.1, (**G**) 849.2, (**H**) 865.2, and (**I**) 1153.3. Scale bar = 5 mm.

It has been reported that several proanthocyanidins, namely B-type procyanidin and propelargonidin oligomers, and their flavan-3-ol monomers are present in strawberry fruit [31,32]. Next, to investigate whether proanthocyanidins and their monomers can be detected in strawberry fruit sections, we prepared the fruit sections using carboxymethyl cellulose (CMC)-freeze embedding [24] and performed MALDI-MSI analyses in the negative-ion mode, using DAN as a matrix. Figure 2A shows the optical image of strawberry section before MALDI-MSI. Five tissues, namely the skin, cortical tissue, vascular bundles, pith tissue, and calyx were observed. Figure 2B shows a mass spectrum ranging from a *m*/*z* of 240 to 1200 obtained by MALDI-MSI. Regarding flavan-3-ol monomers, peaks at *m*/*z* 273.1 and 289.1 corresponding to (epi)afzelechin and (epi)catechin [M − H]^−^ ions were observed. The chirality of C-3 in flavan-3-ols cannot be differentiated by the MS analysis performed in this study; therefore, (epi)afzelechin represented either afzelechin or epiafzelechin. Similarly, (epi)catechin represented either catechin or epicatechin. Regarding proanthocyanidins, we observed peaks corresponding to *m*/*z* values of B-type procyanidins, namely (epi)catechin dimer, trimer, and tetramer [M − H]^−^ ions, at *m*/*z* 577.1, 865.2, and 1153.3, respectively (Figure 2B). When one (epi)afzelechin was present in proanthocyanidins, the signals of procyanidins were shifted by −16 mass units from the signals of B-type procyanidins, corresponding to the loss of one oxygen atom (Figure 1A). Peaks matching the *m*/*z* values of B-type propelargonidin [M − H]^−^ ions with one (epi)afzelechin unit and one or two (epi)catechin units, i.e., peaks at *m/z* 561.1 and 849.2, respectively, were observed (Figure 2B).

To investigate the distribution of peaks at *m/z* 273.1, 289.1, 561.1, 577.1, 849.2, 865.2, and 1153.3, which are potential components of the flavan-3-ols monomer and oligomer [M − H]^−^ ions in ripe strawberry fruit, we constructed their ion images. As shown in Figure 2C–I, all potential components of flavan-3-ols were mainly distributed in the calyx, in and around the vascular bundles, and in the skin.

### 2.3. Identification of Flavan-3-ols in Strawberry Fruit Sections

**Figure 3:**
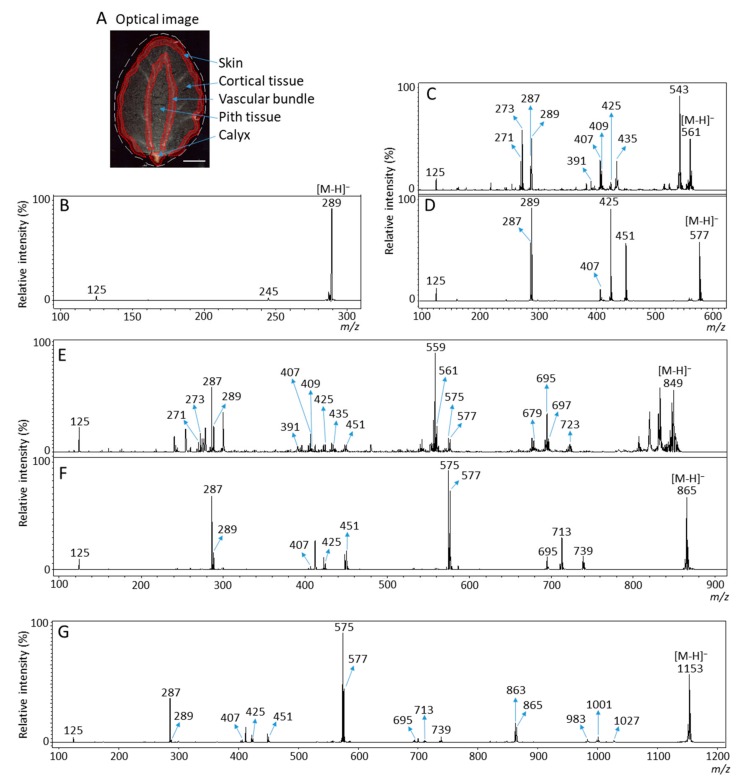
Matrix-assisted laser desorption/ionization (MALDI)-tandem mass spectrometry (MS/MS) analysis of strawberry fruit sections. MALDI-MS/MS spectra were obtained from in the calyx, in and around the vascular bundles, and in the skin of strawberry fruit sections after MALDI-mass spectrometry imaging. (**A**) Optical image of a strawberry fruit section. The dotted red line shows the analyzed region. Scale bar = 5 mm. Representative MS/MS spectra of precursor [M − H]^−^ ions at *m*/*z* (**B**) 289.1, (**C**) 561.1, (**D**) 577.1, (**E**) 849.2, (**F**) 865.2, and (**G**) 1153.3 obtained from a strawberry fruit section. The fragment ion peaks with *m*/*z* values were used for molecular assignments.

MALDI-time of flight (TOF)/TOF analysis enables accurate and highly sensitive elucidation of flavan-3-ols based on their MS/MS spectra [7,8,9]. Next, we performed MALDI-MS/MS analysis to determine whether the observed peaks at *m*/*z* 273.1, 289.1, 561.1, 577.1, 849.2, 865.2, and 1153.3 (Figure 2B) were attributable to flavan-3-ol [M − H]^−^ ions. As shown in Figure 3A, the MS/MS spectra were obtained in the calyx, in and around the vascular bundles, and in the skin of the strawberry fruit section because the potential components of flavan-3-ols were predominantly distributed in these tissues (Figure 2C–I). The MS/MS spectra of potential peaks for flavan-3-ols [M − H]^−^ ions obtained from the three tissues showed similar fragmentation patterns.

MS/MS analysis of the peak of the afzelechin standard [M − H]^−^ ions showed fragment ion peaks at *m*/*z* 257 (loss of water) and 167 (Supplementary Figure S2A). However, in the MS/MS spectra of precursor ion at *m*/*z* 273.1 detected in strawberry sections, the fragment ions at *m*/*z* 257 and 167 were hardly observed (Supplementary Figure S2B). Therefore, we could not determine whether the peak at *m*/*z* 273.1 detected in strawberry sections was attributable to afzelechin and/or epiafzelechin [M − H]^−^ ions. In the MS/MS spectrum of precursor ions at *m/z* 289.1, fragment ions at *m*/*z* 245 and 125 were observed (Figure 3B). This fragmentation pattern agreed with the MS/MS spectra of both catechin and epicatechin standards (Supplementary Figure S2C,D). Therefore, we assigned the peak at *m*/*z* 289.1 in strawberry fruit sections as catechin and/or epicatechin [M − H]^−^ ions.

**Figure 4:**
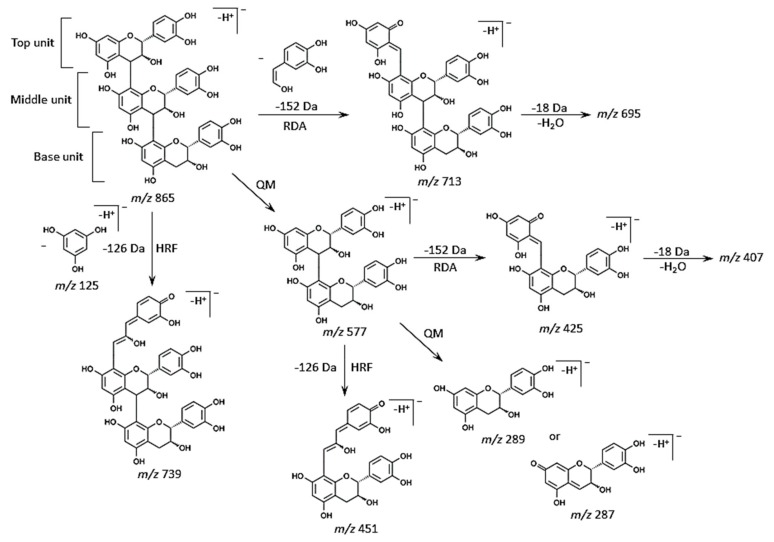
Typical fragmentation patterns of procyanidins by retro-Diels-Alder (RDA), heterocyclic ring fission (HRF), and quinone methide (QM) reactions. Typical fragmentation patterns of B-type procyanidin trimer, (epi)catechin-(epi)catechin-(epi)catechin [M − H]^−^ ion at *m*/*z* 865.

Typical fragmentation patterns of B-type proanthocyanidins observed by liquid chromatography (LC)-MS/MS or MALDI-MS/MS following RDA, HRF, and QM reactions were reported previously [5,6,7,8,9]. For B-type procyanidins composed of (epi)catechin subunits, RDA fragmentation was characterized by the elimination of hydroxyvinylbenzenediol, [M − H − 152]^−^, and loss an additional molecule of water, [M − H − 152 − 18]^−^ ion [8]. For B-type proanthocyanidins, HRF was characterized by the elimination of 1,3,5-trihydroxybenzene, [M − H− 126]^−^ ion [8]. In some cases, deprotonated 1,3,5-trihydroxybenzene was detected at *m*/*z* 125 [8], and this peak was clearly observed in each MS/MS spectrum obtained from the strawberry fruit sections (Figure 3C–G). The MS/MS spectra of precursor ion at *m*/*z* 865.2 are shown in Figure 3F. Figure 4 shows the typical fragmentation pattern of B-type procyanidin, an (epi)catechin trimer. The fragment ion at *m*/*z* 713 reflects RDA reaction of an (epi)catechin unit (−152 Da), and the fragment ion at *m*/*z* 695 corresponding to the loss of a water molecule (−18 Da). The fragment ion at *m*/*z* 739 was caused by HRF reaction of the upper (epi)catechin unit (−126 Da). The fragment ion at *m*/*z* 577 corresponds to the (epi)catechin dimer and is known to originate from QM reaction. Again, the fragment ion of the dimer was observed following the RDA-based reaction (*m*/*z* 425), which yielded ions at *m*/*z* 407 owing to the subsequent loss of a water molecule (−18 Da). The HRF reaction was also observed for the dimer, generating ions at *m*/*z* 451. Finally, a QM reaction with the dimer formed a single quinone, resulting in two fragment ions at *m*/*z* 289 and 287. In addition, the fragmentation pattern of *m*/*z* 865 in strawberry sections was similar to that of the procyanidin C1 standard (Figure 3F, Supplementary Figure S2G). Therefore, we assigned the peak at *m*/*z* 865.2 as B-type (epi)catechin trimer [M − H]^−^, and the sequence as (epi)catechin-(epi)catechin-(epi)catechin (Table 1). MS/MS analysis of the other potential peaks of B-type procyanidins at *m*/*z* 577.1 and 1153.1 clearly showed fragment ions with *m*/*z* values matching the expected ions following RDA, HRF, and QM reactions (Figure 3D,G, Table 1). In addition, the fragment ions of *m*/*z* 577.1, and 1153.3 observed in strawberry sections were similar to those of the procyanidin B1 and B2, and cinnamtannin A2 standards, respectively (Figure 3D,G, Supplementary Figure S2E,F,H). Therefore, we assigned the peaks at *m*/*z* 577.1 and 1153.3 as B-type procyanidin dimer and tetramer [M − H]^−^ ions, and their sequences as (epi)catechin-(epi)catechin and (epi)catechin-(epi)catechin-(epi)catechin-(epi)catechin, respectively (Table 1).

In the MS/MS spectra of the precursor ions at *m*/*z* 561.1 and 849.2, a fragment ion at *m*/*z* 273 matching (epi)afzelechin [M − H]^−^ ion was also observed in addition to the (epi)catechin [M − H]^−^ ion, *m*/*z* 289, by QM reaction (Figure 3C,E, Table 1). It is possible that (epi)afzelechin was present in each unit of B-type propelargonidin dimer and trimer. In the MS/MS spectrum of *m*/*z* 561.1, we observed neutral losses of 136 (*m*/*z* 425) and 152 Da (*m*/*z* 409), generated from the precursor ion by RDA reaction (Figure 3C and Figure 4, Table 1). Elimination of ring B from the flavan-3-ol moiety through an RDA reaction with ring C led to the formation of this ion in previous studies [5,6,7,8,9] (Figure 4). The RDA reaction can occur with either the top or base unit. However, elimination of ring B from the top unit gives rise to a fragment ion with a larger π–π-hyper-conjugated system than RDA-based elimination from the base unit of the dimer [5,6]. Hence, it is more energetically favorable. A loss of 136 Da indicated that ring B of the top unit has a single hydroxyl group, whereas a loss of 152 Da indicated that ring B of the top unit has two hydroxyl groups (Figure 1A and Figure 4). A neutral loss of 126 Da (*m*/*z* 435) by HFR reaction was also observed from the precursor ion (Figure 3C, Table 1). Therefore, we determined the peak at *m*/*z* 561.1 to be a B-type propelargonidin dimer [M − H]^−^ ion with one (epi)afzelechin unit, and the sequences as both (epi)afzelechin-(epi)catechin and (epi)catechin-(epi)afzelechin (Table 1). In the MS/MS spectra of *m*/*z* 849.2 in strawberry fruit sections (Figure 3E), the fragment ions at *m*/*z* 577 and 561 by QM reaction were observed, suggesting that the top unit of B-type propelargonidin trimer was both (epi)afzelechin and (epi)catechin. In addition, we observed neutral losses of 136 Da (*m*/*z* 425) for *m*/*z* 561 and 152 Da (*m*/*z* 409) for *m*/*z* 577 by RDA reaction, suggesting that the middle unit was also both (epi)afzelechin and (epi)catechin (Figure 3E and Figure 4, Table 1). These results suggested that the base unit was also both (epi)afzelechin and (epi)catechin. A neutral loss of 126 Da (*m*/*z* 435 for *m*/*z* 561, and *m*/*z* 451 for *m*/*z* 577) by HFR reaction was also observed (Figure 3E and Figure 4, Table 1). Therefore, the peak at *m*/*z* 849.2 was assigned as B-type propelargonidin trimer [M − H]^−^ ions with one (epi)catechin unit, and the assigned sequences were (epi)afzelechin-(epi)catechin-(epi)catechin, (epi)catechin-(epi)afzelechin-(epi)catechin, and (epi)catechin-(epi)catechin-(epi)afzelechin (Table 1).

### 2.4. Distribution of Identified Flavan-3-ols in Strawberry Fruit

**Figure 5:**
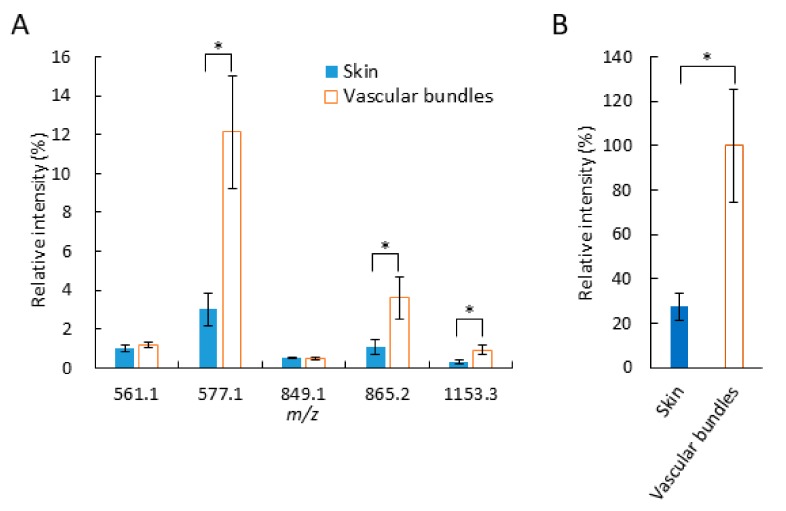
Relative intensities of identified flavan-3-ol species in the skin and in and around the vascular bundles of strawberry fruit sections. Relative intensities of (**A**) *m*/*z* 561.1, 577.1, 849.2, 865.2, and 1153.3, and (**B**) *m*/*z* 289.1. The mean relative intensities of each flavan-3-ol are shown as the relative intensity of the strongest *m*/*z* 289.1, (epi)catechin, in and around the vascular bundle, which was defined as 100%. The data are expressed as mean values ± standard deviation (n = 3). Statistical analyses were performed between in the skin and in and around the vascular bundles of the same flavan-3-ol species. Asterisks (*) indicate significant differences between the mean values in the skin and in and around the vascular bundles, as determined by Mann–Whitney U test at the 5% significance level.

Next, we investigated the distribution of identified flavan-3-ols in strawberry fruit section. As shown in Figure 2A,D–I, (epi)catechin, and B-type procyanidins and propelargonidins, were mainly distributed in the calyx, in and around the vascular bundles and in the skin. Interestingly, the distribution patterns of identified proanthocyanidins between in and around the vascular bundles and in the skin appeared to be different (Figure 2D–I). To further analyze, we compared the signal intensities of each identified proanthocyanidin between skin and vascular bundles (Figure 5A). The mean relative intensities of the identified B-type procyanidins in the skin were significantly lower than those in and around the vascular bundles, whereas those of identified B-type propelargonidins showed no significant differences between the skin and vascular bundles (Figure 5A). Catechin and/or epicatechin showed similar distribution patterns, compared with those of B-type procyanidins (Figure 5A,B). These results suggested that catechin and/or epicatechin and B-type procyanidins biosynthesis were lower in the skin than in and around the vascular bundles, whereas B-type propelargonidin biosynthesis were almost equal between the tissues [1,2].

### 2.5. Investigation of In-Source Fragmentation of Flavan-3-ols

**Figure 6:**
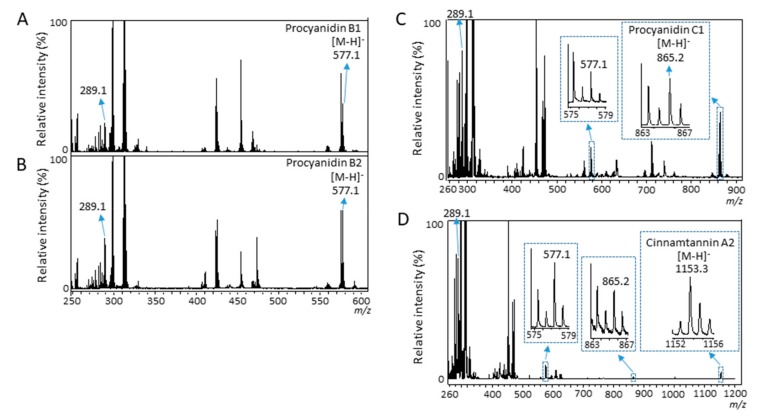
Matrix-assisted laser desorption/ionization-mass spectrometry analysis of procyanidin standards. Mass spectra of (**A**) procyanidin B1, (**B**) procyanidin B2, (**C**) procyanidin C1, and (**D**) cinnamtannin A2 standards. The peaks with *m*/*z* values indicate the peaks matching to identified flavan-3-ols in strawberry fruit sections.

In our previous study using MALDI-MSI analysis of strawberry fruit sections, in-source fragmentations of anthocyanins were suggested by the detection of anthocyanidins [24]. Therefore, to investigate whether in-source fragmentation of proanthocyanidins occurred, we performed MALDI-MS analyses of the procyanidin standards. In the mass spectra of proanthocyanidin dimers, procyanidin B1, and B2 [M − H]^−^ ions, a peak at *m*/*z* 289.1 matching the (epi)catechin [M − H]^−^ ion was clearly observed (Figure 6A,B). In the mass spectrum of procyanidin C1 [M − H]^−^ ion, a peak at *m*/*z* 577.1 matching the (epi)catechin dimer [M − H]^−^ ion was clearly observed in addition to *m*/*z* 289.1 (Figure 6C). Similarly, in the mass spectrum of cinnamtannin A2 [M − H]^−^ ion, a peak at *m*/*z* 865.2 matching the (epi)catechin trimer [M − H]^−^ ion was clearly observed in addition to *m/z* 289.1 and 577.1 (Figure 6D). These results indicated that in-source fragmentation occurred for all proanthocyanidin standards, by some reactions, i.e., QM reactions in the MALDI-MS analysis. These results also suggested that in-source fragmentation occurred for proanthocyanidins in strawberry fruit sections by MALDI-MSI analysis.

## 3. Discussion

In this study, we selected the DAN matrix for analysis after screening various matrices using flavan-3-ol standards and the negative ion mode of MALDI-MSI. We identified (epi)catechin and five proanthocyanidins, namely B-type procyanidin dimer, trimer, and tetramer; and a B-type propelargonidin dimer and trimer with one (epi)afzelechin, using MALDI-MS/MS analysis of ripe strawberry fruit sections. We visualized the identified flavan-3-ols by performing MALDI-MSI analyses, which revealed different distribution patterns between (epi)catechin with B-type procyanidins and B-type propelargonidins in the whole ripe strawberry fruit.

We showed that peaks matching to (epi)catechin monomer or B-type procyanidins were generated from the B-type procyanidins with a larger degree of polymerization by in-source fragmentation (Figure 6A–D). These results suggested that ion images of identified B-type procyanidins, and B-type propelargonidins in the strawberry fruit sections were mixed ion images of itself and fragment ions generated from the B-type procyanidins, and B-type propelargonidins with a larger degree of polymerization (Figure 2D–I). Previous studies [31,32] using LC-MS/MS reported that (epi)afzelechin was located on the top unit of the B-type propelargonidin dimer and trimer with one (epi)afzelechin in strawberry fruit (Figure 5); however, using MALDI-MS/MS, in this study (epi)afzelechin was also located at the base unit of the dimer, and middle or base units of the trimer, respectively (Table 1). This discrepancy might be due to the different strawberry species used here or to the in-source fragmentation of proanthocyanidins. To visualize each flavan-3-ol by itself using MSI, it may be necessary to develop a softer ionization mass-spectrometer than the vacuum MALDI-TOF/TOF used in this study. 

Our data showed that the relative amounts of B-type procyanidins were significantly higher in and around the vascular bundles than in the skin, whereas comparable amounts of B-type propelargonidins were found between in and around the vascular bundles and in the skin (Figure 5A). Although, in the biosynthesis of proanthocyanidins, it has not been fully determined the biosynthesis mechanisms, for example, whether the biosynthesis is enzymatic or not [1], it is known that proanthocyanidins are formed through the condensation of flavan-3-ol units such as (epi)catechin and (epi)afzelechin [1,2]. Procyanidins are biosynthesized from (epi)catechin only, whereas propelargonidins are biosynthesized from both (epi)catechin and (epi)afzelechin [1]. As shown in Figure 5B, the amount of (epi)catechin in and around the vascular bundles was significantly higher than that in the skin. Based on these results, we speculated that the distribution patterns of procyanidins and propelargonidins depended on the distribution patterns of their flavan-3-ol monomers. Therefore, the lack of a significant difference in propelargonidin levels between in the skin and in and around the vascular bundles in strawberry fruit might be caused by smaller differences in the amounts of (epi)afzelechin between in the skin and in and around the vascular bundles, compared with the corresponding (epi)catechin levels.

In angiosperms, flavan-3-ols help protect against ultraviolet radiation and ozone by reducing oxidative stress [1]. Flavan-3-ols can play a key role in the protection against mammals and plant-feeding insects [1]. Tannins have been shown to be toxic to bacteria [1,33]. In addition, flavan-3-ol monomers and proanthocyanidins may also inhibit the germination of fungal spores and block the biosynthesis of melanin, which is an important factor in the virulence of many pathogenic fungi attacking plants [1,34]. Nizioł et al. [35] visualized the only proanthocyanidin, gambiriin C (epiafzelechin-catechin) using MSI with ^109^Ag nanoparticle enhanced target, and showed that the proanthocyanidin was mainly distributed in the skin and achenes of strawberry fruit. They reported that the distribution of flavan-3-ols in strawberry fruit was related to their protective function. In the present study, we revealed that flavan-3-ols are mainly distributed not only in the skin but also in the calyx and in and around the vascular bundles. Pathogens such as bacteria and fungi infect plant surface tissues such as the fruit skin, which proliferate in the infected tissues and immediately spread throughout the plant body through fluid in vascular bundles. Therefore, flavan-3-ols, especially (epi)catechin and B-type procyanidins, might also be present in and around the vascular bundles of ripe strawberry fruit to prevent the infection and spread of pathogens.

Although the biological significance of the different distribution patterns between B-type procyanidins and propelargonidins is currently not well understood, distribution analysis of flavan-3-ols in strawberry fruit under different abiotic and biotic stress conditions will improve our understanding of the biological significance of flavan-3-ols in strawberry fruit [1,2,3]. In addition, the spatial information would assist those working in the field of breeding to enhance the prevention effects of flavan-3-ols against chronic diseases by improving the genetic properties of strawberries [24,36]. Furthermore, the MALDI-MSI analysis used in this study might be adapted to investigate the distribution of flavan-3-ols in various plant species and tissues, except strawberry fruit. Recently, pharmacokinetic analyses, i.e., post-consumption tissue localization analyses of polyphenols such as anthocyanins using MALDI-MSI, have been performed [37]. Therefore, the MALDI-MSI analyses used in this study would be also helpful to investigate the localization of PAs in animal tissues for the elucidation of their health-promoting mechanisms. In conclusion, we demonstrated that the MALDI-MSI analysis used in this study is a valuable tool for distribution analysis of flavan-3-ols in strawberry fruit.

## 4. Materials and Methods

### 4.1. Reagents

(+)-Catechin, (−)-epicatechin, CMC sodium salt, methanol, and water were purchased from Wako Chemicals (Tokyo, Japan). (+)-Afzelechin was purchased from ChemFaces (Wuhan, China). Procyanidin B1 and procyanidin B2 were purchased from Funakoshi Co. (Tokyo, Japan). Procyanidin C1 and cinnamtannin A2 were purchased from Kanto Chemical Co. (Tokyo, Japan). CHCA, 9AA, and DAN were purchased from Tokyo Kasei Co. (Tokyo, Japan). Indium-tin-oxide (ITO)-coated glass slides (100 Ω without MAS coating) were purchased from Matsunami Glass (Osaka, Japan). Peptide calibration stndards containing bradykinin (1–7), angiotensin II, and angiotensin I were purchased from Bruker (Billerica, MA, USA). All reagents and solvents used in this study were of analytical grade.

### 4.2. Strawberry (Fragaria × Ananassa Duch.) Samples

‘Tochiotome’ strawberry fruit, a popular cultivar in Japan, were cultivated in the Strawberry Research Center (Tochigi, Japan). Ten ripe strawberries were harvested, frozen on the day of harvest, and stored at −80 °C until use.

### 4.3. Preparation of Fruit Sections 

Fruit sections were prepared using CMC freeze-embedding, as described in our previous study [24]. Briefly, the fresh fruit were immersed in CMC (2%) and flash-frozen in liquid nitrogen. Subsequently, longitudinal and cross-sections (100 μm thick) were consecutively prepared using a CM 1860 cryostat (Leica Microsystems, Wetzlar, Germany). The sections were then mounted onto ITO-coated glass slides and placed in 50 mL conical centrifuge tubes containing silica gel for drying. The sections were preserved at −80 °C in a freezer until MALDI-MSI analysis was performed.

### 4.4. MALDI-MSI Analysis

MALDI-MSI analysis was performed according to our previous study [24], with minor modifications. The flavan-3-ol standards and strawberry fruit sections were analyzed using a MALDI-TOF/TOF instrument (UltrafleXtreme, Bruker, Billerica, MA, USA) equipped with a 355 nm Nd:YAG laser, using a repetition rate of 1000 Hz. Data were acquired using a step size of 300 μm in negative-ion mode (reflector mode). The *m*/*z* values in the range of 240–1200 were measured. The laser diameter was set to the medium size. The instrument was calibrated externally using the exact *m*/*z* values of CHCA [M − H]^−^ ions (*m*/*z* 188.03532), bradykinin (1–7) [M − H]^−^ ions (*m*/*z* 755.38460), angiotensin II [M − H]^−^ ions (*m*/*z* 1044.52725), and angiotensin I [M − H]^−^ ions (*m*/*z* 1294.67025) as references. The spectra were acquired automatically using FlexImaging 4.1 software (Bruker). Normalization of spectra based on the total ion current was performed using the same software. The FlexImaging 4.1 software was also used to create two-dimensional ion-density maps and for peak analyses.

Matrix screening was performed according to our previous study [25]. Briefly, 1 µL of each standard (10 μg/mL) was mixed with equal volumes of CHCA (10 mg/mL in 70% aqueous methanol), 9AA (10 mg/mL in 70% aqueous methanol), and DAN (10 mg/mL in 80% aqueous methanol) on an ITO-coated glass slide; then, MALDI-MSI analysis was performed.

To analyze the strawberry fruit sections, frozen sections were took out from a freezer and dried in a vacuum desiccator for 30 min. Six milliliters of a DAN solution (10 mg/mL in 80% aqueous methanol) was sprayed uniformly over the section using a 0.18 mm nozzle caliber airbrush (Mr. Airbrush Custom Double Action; Mr. Hobby, Tokyo, Japan), after which MALDI-MSI analysis was performed. To investigate the spatial distribution of the identified proanthocyanidins, three different strawberry fruits were analyzed. The mass spectra and ion images of the identified flavan-3-ols in the three different strawberry fruits showed similar patterns (Supplementary Figure S3). The mass spectrum and ion images of one of the three different strawberry fruits are presented as representative data in Figure 2C–I.

To compare the intensities of the identified flavan-3-ols between the skin and vascular bundles, three sections of the same strawberry fruit were analyzed and their tissue-specific intensities were obtained using the region-of-interest function of FlexImaging 4.1 software. These data are shown in Figure 5.

### 4.5. MALDI-MS/MS Analysis of Strawberry Fruit Sections

MALDI-MS/MS analysis was performed according to our previous study, with minor modifications [24]. Briefly, the MS/MS spectra were obtained from the strawberry sections after performing MALDI-MSI analysis with an ultrafleXtreme instrument operated in collision-induced dissociation “LIFT” MS/MS mode. The *m*/*z* values of precursor ions were set at each value ±1%. The MS/MS spectra were analyzed using FlexAnalysis 3.4 software (Bruker). The molecular species of proanthocyanidins detected from the sections were assigned based on typical neutral losses observed after retro-Diels-Alder (RDA), heterocyclic ring fission (HRF), and quinone methide (QM) reactions [7,8,9], on comparison with the MS/MS spectra of the proanthocyanidin standards.

### 4.6. Statistical Analyses

The data shown in Figure 5A,B are expressed as the mean values and standard deviation (n = 3). Asterisks (*) indicate significant differences in mean values between the skin and vascular bundles, as determined by Mann–Whitney U test at the 5% significance level.

## Figures and Tables

**Table 1:** Flavan-3-ols identified in strawberry fruit sections.

Precursor Ion [M − H]^−^, (*m*/*z*)	Fragment Ions Used for Assignment [M − H]^−^, (*m*/*z*)			Assigned Molecular Species
289.1	245, 125			(Epi)cat
	QM	RDA (RDA-H_2_O)	HRF	
561.1	289, 287, 273, 271	409 (391), 425 (407),	435	(Epi)afz-(epi)cat, (epi)cat-(epi)afz
577.1	289, 287	425 (407)	451	(Epi)cat-(epi)cat
849.2	577, 575 561, 559, 289, 287, 273, 271	697 (679), 425 (407), 409 (391)	723, 451, 435,	(Epi)afz-(epi)cat-(epi)cat, (epi)cat-(epi)afz-(epi)cat, (epi)cat-(epi)cat-(epi)afz
865.2	577, 575, 287, 289	713 (695), 425 (407)	739, 451	(Epi)cat-(epi)cat-(epi)cat
1153.3	865, 863, 577, 575, 289, 287	1001 (983), 713 (695), 425 (407)	1027, 739, 451,	(Epi)cat-(epi)cat-(epi)cat-(epi)cat

Matrix-assisted laser desorption/ionization-tandem mass spectrometry (MALDI-MS/MS) spectra were obtained from strawberry sections after MALDI-MS imaging analysis using an UltrafleXtreme instrument, operated in collision-induced dissociation “LIFT” MS/MS mode. Fragment ions by quinone methide (QM), retro-Diels-Alder (RDA), and heterocyclic ring fission (HRF) were used for molecular assignments. (Epi)cat represents either catechin or epicatechin. (Epi)afz represents either afzelechin or epiafzelechin.

## Supplementary Materials

The following are available online: Figure S1. Mass spectra of flavan-3-ol standards by matrix-assisted laser desorption/ionization-mass spectrometry imaging. Catechin, epicatechin, procyanidin B1, procyanidin B2, procyanidin C1, and cinnamtannin A2 standards were measured using 9-aminoacridine (9AA), α-cyano-4-hydroxycinnamic acid (CHCA), and 1,5-diaminonaphthalene (DAN) in the negative ion mode. The standards were detected as the [M-H]^-^ ion. Red dotted lines indicate each frava-3-ol standard peak. Figure S2. Matrix-assisted laser desorption/ionization-tandem mass spectrometry (MS/MS) analyses of flavan-3-ol standards. Representative MS/MS spectra of the (A) afzelechin standard [M − H]^−^ ion, (B) precursor ion at *m*/*z* 273 with a strawberry section, (C) catechin standard [M − H]^−^ ion, (D) epicatechin standard [M − H]^−^ ion, (E) procyanidin B1 standard [M − H]^−^ ion, (F) procyanidin B2 standard [M − H]^−^ ion, (G) procyanidin C1 standard [M − H]^−^ ion, and (H) cinnamtannin A2 standard [M − H]^−^ ion. MS/MS spectra were obtained using ultrafleXtreme instrument operated in the collision-induced dissociation “LIFT” MS/MS. Figure S3. Representative ion images of the identified flavan-3-ol species in strawberry fruit by matrix-assisted laser desorption/ionization-mass spectrometry imaging. These ion images were obtained from two strawberry fruits different from that shown in Figure 2. The dotted white line shows the analyzed region. Scale bar = 5 mm.
