# Supplementary material for: Distribution of Flavan-3-ol Species in Ripe Strawberry Fruit Revealed by Matrix-Assisted Laser Desorption/Ionization-Mass Spectrometry Imaging
Source: Molecules. 2019 Dec 26;25(1):103. doi: 10.3390/molecules25010103 (PMC6982903; doi:10.3390/molecules25010103)
Supplement: Supplementary file 1 [file molecules-25-00103-s001.pdf]

# Supplementary Materials

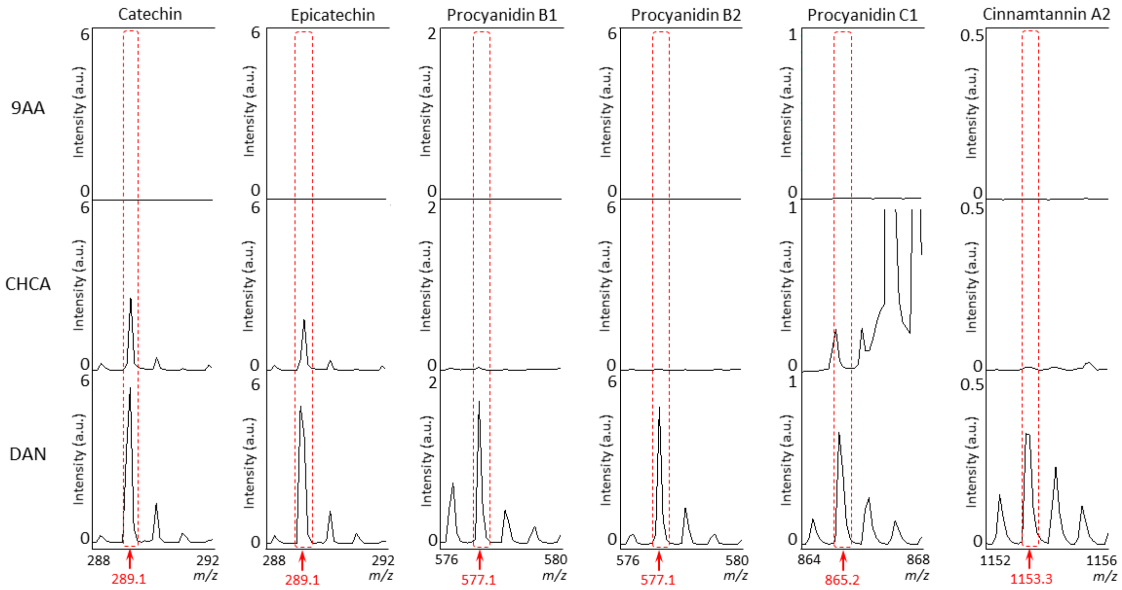


**Figure S1.** Mass spectra of flavan-3-ol standards by matrix-assisted laser desorption/ionization-mass spectrometry imaging. Catechin, epicatechin, procyanidin B1, procyanidin B2, procyanidin C1, and cinnamtannin A2 standards were measured using 9-aminoacridine (9AA), $\alpha$-cyano-4-hydroxycinnamic acid (CHCA), and 1,5-diaminonaphthalene (DAN) in the negative ion mode. The standards were detected as the $[M - H]^-$ ion. Red dotted lines indicate each frava-3-ol standard peak.

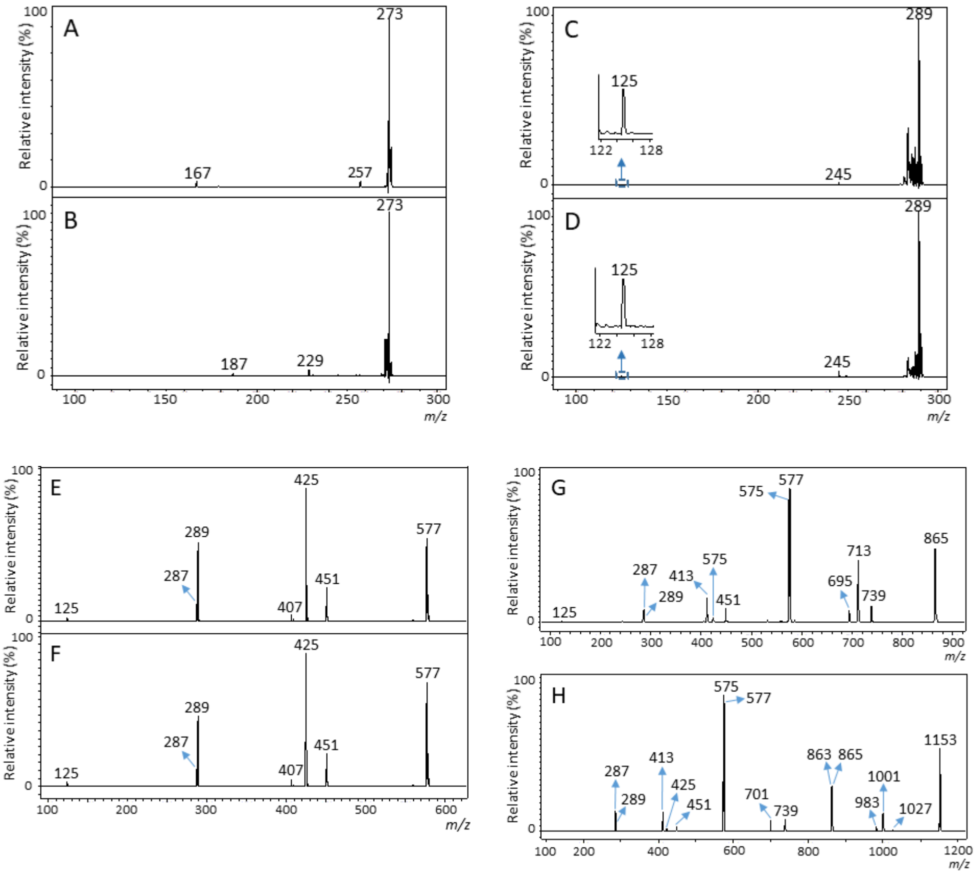


**Figure S2.** Matrix-assisted laser desorption/ionization-tandem mass spectrometry (MS/MS) analyses of flavan-3-ol standards. Representative MS/MS spectra of the (A) afzelechin standard [M − H]⁻ ion, (B) precursor ion at *m*/*z* 273 with a strawberry section, (C) catechin standard [M − H]⁻ ion, (D) epicatechin standard [M − H]⁻ ion, (E) procyanidin B1 standard [M − H]⁻ ion, (F) procyanidin B2 standard [M − H]⁻ ion, (G) procyanidin C1 standard [M − H]⁻ ion, and (H) cinnamtannin A2 standard [M − H]⁻ ion. MS/MS spectra were obtained using ultrafleXtreme instrument operated in the collision-induced dissociation "LIFT" MS/MS.

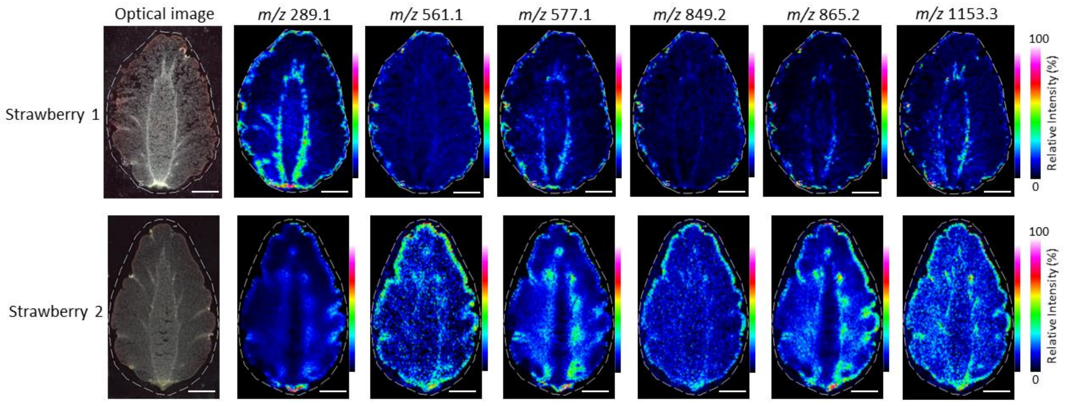


**Figure S3.** Representative ion images of the identified flavan-3-ol species in strawberry fruit by matrix-assisted laser desorption/ionization-mass spectrometry imaging. These ion images were obtained from two strawberry fruits different from that shown in Figure 2. The dotted white line shows the analyzed region. Scale bar = 5 mm.
